# Farmers’ Appraisal on Okra [*Abelmoschus esculentus* (L.)] Production and Phenotypic Characterization: A Synergistic Approach for Improvement

**DOI:** 10.3389/fpls.2022.787577

**Published:** 2022-03-24

**Authors:** Dorcas Olubunmi Ibitoye, Adesike Oladoyin Kolawole

**Affiliations:** ^1^Genetic Resources Unit, National Horticultural Research Institute, Ibadan, Nigeria; ^2^Department of Crop Production and Soil Science, Ladoke Akintola University of Technology, Ogbomoso, Nigeria

**Keywords:** evaluation, farmers’ perception, germplasm, selection, variation

## Abstract

Okra [*Abelmoschus esculentus* (L.) Moench] is a nutrient-rich economically important vegetable crop grown in tropical and sub-tropical regions of the world. Okra is one of the horticultural mandate crops of the National Horticultural Research (NIHORT), Ibadan, Nigeria. It is an under-studied crop in terms of genetic improvement in Nigeria. In response to farmers’ demand for improved varieties, the institute commenced efforts in developing novel okra varieties. However, a successful and sustainable crop improvement program depends on the identification of market-driven demands and the availability of variation in the crop germplasm. In view of the above, this research began with an appraisal study to assess the current situation of okra production and to identify farmers’ preferred traits for establishing breeding priorities. According to the results of the participatory rural appraisal, some of the important constraints affecting okra productivity are lack of improved varieties, diseases, pests, and drought. The quest to assess variability in the collected okra accessions and select superior varieties with farmers’ preferred traits, fifty (50) okra accessions were evaluated in the field for 3 consecutive years (2019–2021) using a 5 × 10 α-lattice incomplete block design with three replications. The ANOVA revealed high significant variation (*p* < 0.001) in the number of days to first flower, pod length, and pod girth. The variability observed among the accessions will be useful in the selection of potential parents required for hybridization and generation of progenies with desirable traits for farmers.

## Introduction

Cultivated okra (*Abelmoschus esculentus* (L.) Moench] is an important annual fruit vegetable commonly grown in the tropics and warmer temperate regions of the world (Patil et al., 2015). Okra is generally a self-pollinating crop belonging to the Malvaceae (Oppong-Sekyere et al., 2011). Okra is a multipurpose and economic crop for farmers and marketers in Nigeria because of the income generated from the sale of immature fresh leaves, fresh, and dried fruits, which are made into diverse soup products. It is a nutritious food with many health benefits. It is rich in dietary fiber, minerals (Sodium, Calcium, Sodium, Potassium, Zinc, and Iron), vitamins (A, B, and C), antioxidants, and folate. Okra seed is rich in proteins (15–26%), the seed oil is edible (20–40%), and rich in unsaturated fatty acids like the linoleic acid essential for human nutrition. The mature fruit and stems are used in the paper industry. Okra mucilage can be used as food additives (NRCNAs, 2006; Benchasri, 2012; Kumar et al., 2013, 2017; Gemede et al., 2015; Dubey and Mishra, 2017).

The global okra production is estimated to be around 9.96 million tons, India leading with 6.18 million tons followed by Nigeria with 1.82 million tons (FAOSTAT, 2020). Nigeria recorded the highest okra fruit yield in 2010 with an average yield of 27,275 kg/ha, and since then it has been on the decline with the sharpest drop recorded in 2011 with an average yield of 8,735 kg/ha. The complex interactions of biotic and abiotic stresses are implicated in the decrease in fruit yield over the years. The scarcity of improved okra varieties with important desirable end-user traits also contributes to the yield decline (Kumar et al., 2010).

There are previous studies on the characterization of okra germplasm with the aim of improvement (Komolafe et al., 2021). However, these studies do not consider farmers’ input for desirable traits. Lack of this important information from farmers’ perspectives may have led to low adoption of improved okra varieties in Nigeria. Breeding for improved cultivars is based on selection for traits, such as yield, resistance to pests and diseases, good adaptation, and value-added traits. However, the final marketable product is an integration of several desirable traits that can only be identified through a market-driven breeding approach, which enables the involvement of stakeholders along the value chain in the varietal development (Reddy et al., 2016). With this approach, the breeder has the opportunity to select improved products with customer-centered traits, which invariably boosts farmers’ confidence in the product and its acceptability. Success of market-driven approach, however, is achieved through the inclusion of market-desired traits and the availability of information about the nature and degree of genetic diversity of the traits among the crop germplasm. The participatory rural appraisal (PRA) is one of the methods for identifying market-driven traits. This method is useful for breeders to identify farmers’ constraints and their preferred traits in an improved variety. It is designed to bridge the gap between breeders and other stakeholders along the value chain to ensure that the new varieties satisfy stakeholders’ preferences and suit their socio-economic condition (Kiiza et al., 2012; Sandham et al., 2019).

This study was designed to identify the constraints to okra production, identify farmers’ preferred traits using the PRA technique, and to evaluate the existing okra germplasm for variability and selection of superior lines that can be potential parents for designing customer-driven okra varieties.

## Materials and Methods

### Participatory Rural Appraisal

Using multi-stage sampling approach, a semi-structured survey questionnaire and focus group discussion (FGD) were used to collect information in the selected areas. The first stage is the purposive sampling of the local government areas (LGAs) based on okra production as revealed by the local agricultural extension agency. One community was chosen at random from each LGA, and okra farmers were chosen at random from the selected communities, yielding a total of 120 respondents. For the FGDs, one FGD was conducted in each community, with 12 farmers in each group, including male, female, and youth, to assess the gender view to the questions asked (Table 1). Data collected from the FGD were used to validate the responses obtained from the individual interview.

**TABLE 1:** Study areas and distribution of farmers for interview and FGD.

Community	Coordinates		*Elevation (masl)	LGA	No of farmers	
	*N*	*E*			Interviews	FGD
Sobi Barracks	8.55	4.56	261	Ilorin South	12	12
Ogundele	8.49	4.45	304	Ilorin West	22	12
Aigoro	8.67	4.88	289	Ilorin East	26	12
Balla	8.40	4.42	359	Asa	30	12
Yeregi	8.75	4.48	311	Moro	30	12
Total					120	60

*masl, meter above sea level; LGA, Local Government Area; FGD, focus group discussion.

Socio-demographic data was first collected from the respondents on age, gender, marital status, educational level, primary occupation, years of farming experience, farm size, land ownership, okra cultivation per year and type of cultivation system used for okra production, okra variety grown, and sources of seeds grown (Supplementary Table 1). They were then asked to rank the extent to which certain factors (lack of improved cultivars, drought, susceptibility to pests and diseases, gender, and land ownership) limits their productivity (Great extent = 1, some extent = 2, little extent = 3, no extent = 4). Finally, respondents were asked to rank their choice/preference of traits to be selected for in improved okra varieties in order of importance (very important = 1, important = 2, and not important = 3). Later respondents were asked about their opinion about participatory breeding. The interview was conducted using the local language (Yoruba) and the interviewees were divided into male and female groups to establish easy communication. Participants were clearly informed about the purpose of the study, the questions, and the use of the result of the study. All respondents voluntarily gave their verbal consent to participate in the study.

### Phenotypic Diversity in Okra Germplasm and Its Usefulness in Okra Improvement

A total of 50 okra accessions from the genebank of National Horticultural Research Institute (NIHORT), Ibadan were evaluated in 3 different environments: (i) the vegetable research field of NIHORT during the wet season of 2019 [N7.40287°; E3.84856°; 162.46 m above sea level (masl)]; (ii) Teaching and Research Farm, University of Ilorin, dry season of 2020 (N8.4928°; E4.5962° 350 masl); and (iii) wet season of 2021 (N7.40160°; E3.84804°; 166.84 masl) giving 3 independent environments. The accessions were evaluated using a 5 × 10 α lattice design with three replications on a plot of size 5.4 m^2^ (3 m × 1.8 m), the plants were planted on two rows on the plot. The plants were at 0.5 m intra-row spacing. Regular and necessary cultural practices were carried out as and when required. Owing to the variability in the weather conditions at the different seasons and years of evaluation, only 22 accessions with complete data across all the test environments were used in this study.

### Agronomic Data

The data collected were based on the descriptor by the International Plant Genetic Resources (IPGRI, 1991) for okra. Data were recorded on five randomly selected and tagged plants for each genotype in a replicate on number of days to first flower (DFF), plant height (HT), stem girth (SG), number of pods per plant (PODPLT), pod length (PODLT), pod girth (PODGTH), pod weight (PODWT), stem color (SC), petal color (PC), leaf color (LC), fruit color (FC), and fruit pubescence (FP).

### Data Analyses

Statistical Package for Social Science (SPSS (Statistical package for social sciences), 2007 SPSS version. 20) software was used to analyze the PRA data. Descriptive statistics were used to determine the frequencies and percentages of respondents’ replies to the collected data. ANOVA was computed for data collected on quantitative traits using PROC GLM in SAS (SAS Institute, 2010) considering genotypes as fixed effect and replication and environment as random effects to determine significant variations among measured traits. At 0.05 probability level, means of traits with significant variation were separated using the Least Significant Difference (LSD). Multivariate analysis was computed using the standardized mean values for the data. Principal component analysis (PCA) was computed to determine the percentage contribution of each trait to the observed total variability among the studied okra accessions. Principal components with eigen values > 1.0 were selected to indicate traits with significant contribution (Jeffers, 1967; Ibitoye et al., 2020).

The PROC Cluster and PROC TREE were used to generate dendrogram to show the relationship between the okra accessions studied. Pearson correlation was performed using PROC CORR function in SAS to determine the relationship among the measured traits. A rank summation index (RSI) (Mulumba and Mock, 1978; Kolawole et al., 2021) was constructed to create the aggregate trait by ranking accessions based on number of days to first flower, stem girth, and pod girth. Ranks were summed for each accession to select the top and bottom five. Selection differential (%) was calculated as the proportion of mean for all accessions.

## Results

### Participatory Rural Appraisal

#### Socio-Demographic Data of Respondents

From the survey data (Supplementary Table 1), the majority of respondents were male (73%). The majority of farmers were between the age of 31 and 60 years (71%), 23% were less than 30 years while 7% were above 60 years. This survey revealed a large gender gap with 73% of respondents being male and 27% being the women. A larger percentage (89%) of the respondents were married. The majority of the respondents (31%) had no schooling, 24 and 23% had primary education and secondary education, respectively, 10% had tertiary education, and 13% had quranic and adult education. The study communities are predominantly agrarian with a majority (97%) of the respondents having farming as their primary occupation. Most of the respondents carry out their farming activities on inherited land (53%) and cultivate okra at least twice a year (58%). The majority of respondents (78%) use the mixed cropping method, with 93% growing an unimproved landrace of okra. Their major source of getting okra seeds for sowing is seeds preserved from their past harvest (53%).

#### Okra Farmers’ Production Constraints and Preferences

The respondents for the FGD were asked to identify and rank production constraints while individual respondents were asked to rank the constraints in order of extent to which each constraint limits okra production (Table 2). During the FGD, the lack of improved varieties was ranked first in all communities except Yeregi, where it was ranked second. This could be because Yeregi is closer to the city center where seed sellers are easily accessible. Individual respondents were asked about okra production challenges and were asked to rank the problems using extent of limitation each has on okra production. Seventy-five percent of the respondents indicated diseases as constraint to production while 74% of the respondents indicated that insect pests constrain their okra production to a great extent, followed by drought (71%) and lack of improved varieties (67%). Land ownership with respect to gender was not a serious constraint to production (86%).

**TABLE 2:** Okra production constraint rank across community during FGD and individual respondents.

Production constraints	Sobi Barracks	Ogundele	Aigoro	Balla	Yeregi	Mean	*Great extent	Some extent	Little extent
Lack of improved varieties	1	1	1	1	2	1.2	80(66.7)	39(32.5)	1(0.8)
Drought	2	3	1	1	2	1.8	85(70.8)	17(14.2)	18(15)
Diseases	1	1	2	2	2	1.6	90(75)	21(17.5)	9(7.5)
Insect pests	2	1	2	2	1	1.6	89(74.2)	19(15.8)	12(10)
Cost of land rent/lease	4	4	3	4	5	4	58(48.3)	17(14.2)	45(37.5)
Gender with respect to land ownership	5	5	6	4	4	4.8	3(2.5)	14(11.7)	103(85.8)
Poor soil fertility	4	3	5	4	3	3.8	63(52.5)	25(20.8)	32(26.7)

*Percentage in parentheses, FGD, focus group discussion.

In order of importance, farmers were asked to rank must-have traits in improved okra varieties (Table 3). The top five must-have traits are yield (100%), resistance to pest and disease (90%), high market acceptability (85%), earliness (83%), drought tolerance (74%) and spineless (62%).

**TABLE 3:** Preferred traits in improved okra variety.

*N* = 120	Very important	Important	Not important
High yield	120(100)	–	–
High viscosity	70(58.3)	41(31.2)	9(7.5)
Resistance to pest and disease	108(90)	11(9.2)	1(0.8)
Earliness	99(82.5)	15(12.5)	6(5)
Spineless	74(61.7)	36(30)	10(8.3)
High market acceptability	102(85)	15(12.5)	3(2.5)
Drought tolerance	89(74.2)	18(15)	13(10.8)
Large pods	65(54.2)	29(24.2)	26(21.7)

#### Assessment of Farmers’ Perception on Participatory Breeding

Perception of farmers about participatory breeding was assessed. About 93% of the respondents accepted their inclusion at formulation stage, on-farm-trials, evaluation, and selection (97%) (Supplementary Table 2).

### Variation in Agronomic Traits

Okra accessions used in this study varied for the qualitative traits assessed (Table 4). The color of the fruit varies from green, yellowish-green to green with red patches. About 64% of the accessions had smooth fruit. Petal color varied between cream and yellow, while stem color ranged between green and green with red patches. The accessions differed significantly (*P* < 0.05) for number of days to first flower, stem girth and pod girth. There were significant differences among the environments for all traits which suggest that the environments were independent to discrimination among the accessions. There was significant interaction of genotype and the environment for number of days to flowering, stem girth, and pod girth (Table 5).

**TABLE 4:** Variation in qualitative characters among okra genotypes.

Genotype	Stem color	Petal color	Fruit color	Leaf color	Fruit pubescence
NHAB1	Green	Cream	Green	Green	Smooth
NHAB10	Green	Cream	Green	Green	Smooth
NHAB11	Green	Yellow	Yellowish green	Green with red veins	Smooth
NHAB12	Green with red patches	Yellow	Green with red patches	Green with red veins	Slightly rough
NHAB13	Green with red patches	Yellow	Green	Green with red veins	Prickly
NHAB14	Green with red patches	Yellow	Yellowish green	Green with red veins	Slightly rough
NHAB15	Green with red patches	Cream	Green	Green with red veins	Slightly rough
NHAB16	Green	Cream	Green	Green	Smooth
NHAB17	Green with red patches	Yellow	Green	Green with red veins	Prickly
NHAB18	Green	Cream	Green	Green	Smooth
NHAB19	Green with red patches	Yellow	Green	Green with red veins	Smooth
NHAB2	Green	Cream	Green	Green	Slightly rough
NHAB20	Green with red patches	Yellow	Green with red patches	Green with red veins	Downy
NHAB21	Green with red patches	Yellow	Green with red patches	Green with red veins	Slightly rough
NHAB22	Green	Cream	Green	Green	Smooth
NHAB3	Green	Yellow	Green	Green	Smooth
NHAB4	Green	Cream	Yellowish green	Green	Slightly rough
NHAB5	Green	Cream	Green	Green	Smooth
NHAB6	Green with red patches	Yellow	Yellowish green	Green with red veins	Smooth
NHAB7	Green	Cream	Green	Green	Smooth
NHAB8	Green with red patches	Yellow	Yellowish green	Green with red veins	Smooth
NHAB9	Green with red patches	Yellow	Green	Green with red veins	Smooth

**TABLE 5:** Mean squares from ANOVA for agronomic characters.

Source	*DF	DFF	HT	SG	PODPLT	PODGTH	PODPLT	PODWT
Environment (env)	2	986.53***	8228.15***	24818.91***	38934.45***	4643.72***	8598.27***	226658.39***
Replication × env	6	10.79	491.17	42.34	22.33	0.77	252.97**	10180.37**
Genotype	21	61.85***	532.43	605.76***	59.79	4.69***	70.2	1824.68
Genotype × env	42	44.35***	506.91	362.58***	60.65*	4.06***	89.93	2162.39
Error	126	7.82	404.92	132.65	41.35	1.88	81.13	2172.38
CV (%)		5.68	97.79	44.09	34.79	16.24	70.57	73.48

*, **, ***Significant at 0.05, 0.01, and 0.001 probability levels, respectively.

*DF, degree of freedom; DFF, no of days to first flower (days); HT, plant height (cm); SG, stem girth (g); PODPLT, pod per plant; PODGTH, pod girth (mm); PODWT, pod weight (g).*

Mean performance of the okra accessions is presented in Table 6. Variation observed in number of days from sowing to first flower ranged between 44.94 and 54.14 days with a mean of 49.14 days. Accession NHAB6 was the earliest to flower (44.94 days). Pod length also varied among the accessions between 14.93 and 233.86 with NHAB9 having the longest pod among the accessions. Number of pods per plant ranged between 33.96 for NHAB3 and 86.02 for NHAB18.

**TABLE 6:** Mean performance of agronomic traits of okra accessions.

Accession	DFF	HT	SG	PODLT	PODGTH	PODPLT	PODWT
NHAB1	51.53	18.30	19.62	15.69	7.75	13.00	61.36
NHAB2	52.72	16.26	21.67	20.95	8.02	10.83	49.74
NHAB3	52.72	50.60	20.19	19.76	8.45	7.86	33.96
NHAB4	47.33	17.33	34.76	17.70	8.02	8.81	41.57
NHAB5	47.92	23.49	37.56	15.96	8.51	13.56	75.19
NHAB6	49.11	20.09	29.89	18.08	8.42	10.33	51.13
NHAB7	44.94	16.29	27.85	16.88	8.68	17.22	77.76
NHAB8	46.50	15.79	22.31	20.41	8.98	11.78	69.77
NHAB9	54.14	28.85	26.04	14.96	8.68	18.17	84.71
NHAB10	51.56	17.47	19.23	16.23	10.09	12.08	60.94
NHAB11	46.64	14.47	19.87	14.93	6.99	10.25	52.53
NHAB12	48.31	15.49	23.65	21.19	8.20	13.03	54.89
NHAB13	47.72	17.52	22.16	19.00	8.79	12.89	67.11
NHAB14	52.94	23.68	24.33	15.45	8.20	11.28	58.50
NHAB15	47.75	16.90	19.82	20.49	9.11	13.50	63.43
NHAB16	50.33	21.76	24.08	15.91	7.83	11.36	58.57
NHAB17	49.78	24.50	54.03	22.51	9.09	14.25	82.08
NHAB18	46.39	17.34	23.83	20.01	8.26	17.97	86.02
NHAB19	49.22	16.48	25.57	19.17	7.68	15.11	77.31
NHAB20	50.56	24.79	35.52	20.33	9.85	15.86	79.43
NHAB21	50.33	19.12	22.82	17.14	8.36	11.19	52.01
NHAB22	45.28	16.19	19.83	23.86	7.54	10.47	57.47
Minimum	44.94	14.47	19.23	14.93	6.99	7.86	33.96
Maximum	54.14	50.60	54.03	23.86	10.09	18.17	86.02
Mean	49.26	20.58	26.12	18.48	8.43	12.76	63.43
LSD (0.05)	2.61	18.77	10.74	5.99	1.28	8.40	43.48
SE ±	0.93	6.71	3.84	2.14	0.46	3.00	15.54

*DFF, no of days to first flower (days); HT, plant height (cm); SG, stem girth (g); PODPLT, pod per plant; PODGTH, pod girth (mm); PODWT, pod weight (g).*

Pearson correlation (*r*) analysis was used to determine the relationship between the measured characters (Table 7). There was a positively strong and significant correlation between number of pods per plant and pod weight per plant (*r* = 0.92***). Plant height and number of days to first flower showed a significant correlation (*r* = 0.54*).

**TABLE 7:** Correlation coefficient (*r*) between pairs of measured characters.

	Plant height (cm)	Stem girth (mm)	Pod length (cm)	Pod girth (mm)	Pod/plant	Pod weight (g)
Days to first flower (day)	0.54*	–0.04	–0.30	0.18	–0.08	–0.16
Plant height (cm)		0.12	–0.03	0.17	–0.18	–0.21
Stem girth (mm)			0.17	0.28	0.23	0.39
Pod length (cm)				0.13	–0.04	0.03
Pod girth (mm)					0.31	0.35
Pod/plant						0.92***

*, ***Significant at 0.05 and 0.001 probability levels, respectively.

**FIGURE 1:**
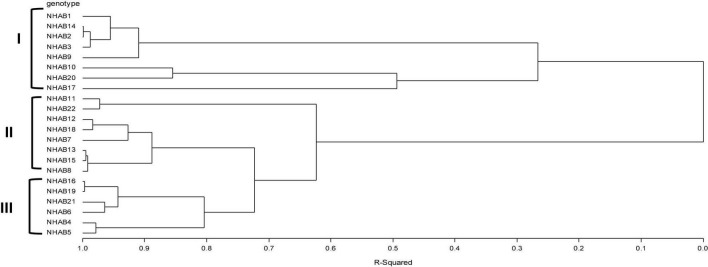
Multivariate cluster analysis using Ward’s minimum variance method showing relatedness of okra accession.

Cluster analysis was performed on the accessions to assess the extent of relatedness among them. Characters showing significance in the analysis of variance were used as discriminant variables for the cluster analysis. Cluster analysis based on Ward distance grouped the okra accessions into three main clusters with sub-groups within the main clusters (Figure 1). Clusters I and II had 8 accessions each, while cluster III had 6 accessions.

The RSI of the 22 okra accessions identified NHAB15, NHAB18, NHAB20, NHAB13, and NHAB17 as outstanding in performance. These five accessions have a combination of excellent agronomic traits. Okra accession NHAB20 was superior in terms of stem and pod girth and NHAB18 had the least number of days to flowering (Table 8).

**TABLE 8:** Rank performance of outstanding okra accessions.

Genotype	DFF	SG	PODGTH	Rank
**Top 5**				
NHAB15	47.75	25.57	9.11	19
NHAB18	46.39	29.89	8.26	21
NHAB20	50.56	35.52	9.85	21
NHAB13	47.72	24.08	8.79	23
NHAB17	49.78	27.85	9.09	23
Mean of top 5	48.44	28.58	9.02	
Grand mean	49.26	26.12	8.43	
Standard error (±)	0.93	3.84	0.46	
Selection differential (%)	−1.67	9.42	6.99	
**Bottom 5**				
NHAB9	54.14	22.31	8.68	44
NHAB14	52.94	24.33	8.20	45
NHAB3	52.72	19.82	8.45	50
NHAB2	52.72	19.62	8.02	57
NHAB1	51.53	19.23	7.75	58
Mean of bottom 5	52.81	21.06	8.22	

*DFF, no of days to first flower (days); SG, stem girth (mm); PODGTH, pod girth (mm).*

Principal component analysis showed the contribution of each trait to the total variation observed. The first three PCA with eigenvalues > 1 accounted for 75% of the variation. PCA1 accounted for around 34% of the variation, PCA2 for 24%, and PCA3 for 17% (Table 9). In PCA1 traits, which had a high and positive contribution, were number of pods/plants (0.58) and pod weight (0.62). The PCA2 was mainly loaded with days to first flower (0.63), plant height (0.61), and pod girth (0.39). Stem girth (0.39), and pod length (0.77) had positive contributions in PCA3.

**TABLE 9:** Eigen values and percentage of total variation among okra accessions by the first three principal component.

	PC axes		
	Prin1	Prin2	Prin3
Eigen values	2.36	1.70	1.21
Explained proportion of variance (%)	34	24	17
Cumulative proportion of variance (%)	34	58	75

**Character**	**Eigen vectors**		

Days to first flower (day)	−0.16	**0.63**	−0.21
Plant height (cm)	−0.17	**0.61**	0.20
Stem girth (mm)	**0.34**	**0.20**	**0.39**
Pod length (cm)	0.11	−0.18	**0.77**
Pod girth (mm)	**0.33**	**0.39**	0.22
Number of pods/plants	**0.58**	0.04	−0.30
Pod weight (g)	**0.62**	0.02	−0.19

*Only eigenvectors with values ≥ 0.30 which largely controlled each PC axes are boldfaced.*

## Discussion

### Socio-Economic Characteristics of Respondents

Preponderance of male farmers across the surveyed communities is based on the cultural and religious beliefs. The majority of women works with their husbands on the farm and are not involved much in physical farm activities but are more involved with the harvest and post-harvest activities. This agrees with the findings of Emokaro et al. (2007). The majority of respondents were married with a relatively large household. According to the farmers, the large household size is very important as it provides easy access and inexpensive labor for their farming activities. The majority of the farmers had no formal education. This result is similar to the findings of Nwaobiala and Nwosu (2013) and Mohammed et al. (2021). A large percentage of the respondents have been growing okra for more than 26 years. This means that they are well experienced in okra production and are used to their production practices, such as intercropping with other crops, like cassava and saving seeds, from previous harvest of local varieties. Mixed cropping is the major cropping system used by the respondents. The farmers argued that mixed cropping help them to maximize their lands and provides multiple sources of income (Mawo et al., 2016; Cobbinah and Kwoseh, 2021).

### Assessment of Production Constraints, Preferences and Participatory Breeding

Identifying farmers’ constraints that limit productivity in a specific target environment is important for breeding programs to be able to develop products that are demand-driven. Understanding their production constraints will give effective direction to the breeding efforts and make adoption of the new varieties easy. In this study, lack of improved okra varieties, susceptibility of the local varieties to pest and diseases and drought were identified as major production constraints. The cultivation of improved varieties will increase farmers’ productivity resulting in higher income and better standard of living for them (Persley and Anthony, 2017; Ume and Ochiaka, 2018).

High yield was ranked as the most preferred trait in improved okra variety. Mohammed et al. (2021) reported a similar choice of high yield among cowpea farmers in Nigeria. Other perceived traits in their order of preference were resistance to insects and diseases, high market acceptability, earliness, and drought tolerance. Participatory appraisal combines methods for identifying problems with the stakeholders, analyzing their knowledge, and perception and together planning to proffer solutions to the problems (Sandham et al., 2019). The farmer’s perception was assessed on participatory approach to breeding. The majority of respondents mentioned that they do not want to be brought in at the last stage of varietal testing, but rather want to be involved right from the conceptualization and product design stage up to varietal evaluation and selection. The farmers responded that this will enable them to own the final product and therefore, makes adoption very easy and fast.

### Agronomic Evaluation of Okra Accessions

The study showed the variation among the morphological traits of the okra accessions. The three different environments used for this study showed variability in the performance of the accessions for all traits measured. During the phases of okra growth, the edaphic and climatic condition had played a significant role in their performance. However, the significant mean squares attributed to genotype x environment interaction and genotype were only for a number of days to first flower, stem girth, and pod girth. In spite of the diversity of accessions for these traits, there is a need to test these accessions in additional environments, for consistent performance, which is an important factor for variety adoption by farmers. The variations observed will aid selection for improvement, considering that superior accession with farmer’ preferred trait identified from this study will be used as parental lines for hybridization and other breeding activities. Previous findings reported significant variation for agronomic traits in okra (Ariyo and Odulaja, 1991; Vandana et al., 2014; Komolafe et al., 2021).

The numbers of days to flowering in this study were lower than those reported by Alake (2019). In this study, an accession (NHAB7), which flowered 45 days after planting, was identified while the late flowering accession (NHAB9) flowered 54 days after planting. This variability thus provides the opportunity for breeding for earliness, which will be useful for double-cropping or relay-cropping systems in an environment with increased rainfall duration (Mattedi et al., 2015; Assogba et al., 2016). From the PRA carried out in this study, earliness was ranked among the top five traits preferred in improved okra varieties by the farmers. Similarly, the variability observed in stem girth and pod girth which may eventually translate into productivity will boost selection of preferred variety for developing farmers.

Correlations among the characters play an important role during crop improvement and increase the effectiveness of selection (Falconer and Mackay, 1996). Significant positive correlations were observed between the two traits. The strong positive significant relationship between the number of pods and pod weight per plant suggested that as number of pods increases, so does their weight, resulting in increased productivity. Likewise, the number of days to first flower had a positive correlation with plant height. This is an indication that some characters can be indirectly targeted in a selection criterion, especially characters that are costly to measure or easily influenced by the environment (Naveed et al., 2009; Nyadanu et al., 2017; Vrunda et al., 2019).

Significant positive and or negative correlations were observed among several characters. This was an indication that some characters can be indirectly targeted in a selection criterion, especially characters that are costly to measure or easily influenced by the environment (Nyadanu et al., 2017). Therefore, parental lines with desirable traits can be selected from these okra accessions for further hybridization and other breeding activities.

Using RSI to select outstanding accessions is useful for identifying putative parents for hybridization (Ghani et al., 2020; Ibitoye et al., 2020). The rank performance identified accessions NHAB15, NHAB18, NHAB20, NHAB13, and NHAB17 as superior for number of days to first flower, stem girth, and pod girth. The RSI showed that NHAB15 ranked best and NHAB17 ranked 5th, although NHAB18 which ranked 2nd flowered earlier and gave the highest pod weight across the environments. Also, in the second rank (RSI = 21) was NHAB20 which combines stem girth and pod girth. Thus, the selection of these accessions for further improvement will enhance productivity.

Furthermore, the PCA compliments the results from ANOVA and correlation as number of pods per plant, pod weight, number of days to the first flower, plant height, pod girth, stem girth, and pod length explained the entire phenotypic variability among the accessions. The okra accessions were grouped based on similarities for one or more morphology traits. Cluster I consisted of 8 accessions which are late flowering ranging between 50 (NHAB17) and 54 days (NHAB9) after planting. This cluster had the accession (NHAB17) with the widest stem girth (54 mm), accession (NHAB10) with the widest pod girth (10.1 mm). The early maturing accessions were grouped together in cluster II, NHAB7 was the earliest to flower (45 days after planting). This accession also had the widest stem girth (27.85 mm) in the cluster. Cluster III had the intermediate flowering group ranging between 47 (NHAB4) and 50 days for accession (NHAB21). NHAB5 had the widest stem girth (37.56 mm) and widest pod girth (8.51 mm) in the cluster. The cluster analysis aids the identification of genetically diverse parents for hybridization. Crossing of accessions from different clusters will express heterosis, and produce desired segregants (Ahiakpa et al., 2013).

## Conclusion

The inclusion of farmers’ production constraints and preferred traits in designing breeding programs will enhance adoption of improved varieties and increase productivity. The observed variability among the accessions evaluated in this study provides a strong basis for selecting farmer’s preferred traits.

In this study, the prevailing cropping system for okra farmers’ production constraints and preferred traits were identified. Most of the interviewed farmers ranked lack of improved varieties and susceptibility of available varieties to pests and diseases as major constraints limiting productivity. The result from PRA revealed that farmers ranked earliness among the top five preferred traits. Some early flowering accessions (NHAB7 and NHAB22) were identified in this study which can serve as potential parents for the development of early okra lines. The result from phenotypic evaluation identified number of pods per plant, pod weight, number of days to first flower, plant height, pod girth, stem girth, and pod length contribute significantly to the variability among the accessions. This variability gives a strong basis for selection of farmer preferred traits. Accessions NHAB15, NHAB18, NHAB20, NHAB13, and NHAB17 had outstanding performance and can be useful in the okra improvement program.

## Data Availability Statement

The raw data supporting the conclusions of this article will be made available by the authors, without undue reservation.

## Ethics Statement

The studies involving human participants were reviewed and approved by Vegetable and Floriculture Research Programme, National Horticultural Research Institute, Nigeria. Written informed consent for participation was not required for this study in accordance with the national legislation and the institutional requirements.

## Author Contributions

DI: conceptualized the study. DI and AK contribute to methodology, experimentation, investigation, writing-original draft, reviewing, and editing. Both authors have read and agreed to the published version of the manuscript.

## Conflict of Interest

The authors declare that the research was conducted in the absence of any commercial or financial relationships that could be construed as a potential conflict of interest.

## Publisher’s Note

All claims expressed in this article are solely those of the authors and do not necessarily represent those of their affiliated organizations, or those of the publisher, the editors and the reviewers. Any product that may be evaluated in this article, or claim that may be made by its manufacturer, is not guaranteed or endorsed by the publisher.
