# Supplementary material for: Farmers’ Appraisal on Okra [Abelmoschus esculentus (L.)] Production and Phenotypic Characterization: A Synergistic Approach for Improvement
Source: Front Plant Sci. 2022 Mar 24;13:787577. doi: 10.3389/fpls.2022.787577 (PMC8988028; doi:10.3389/fpls.2022.787577)
Supplement: Supplementary file 1 [file Table_1.DOCX]

| Supplementary 1: Socio-economic characteristics and farming history of respondents | | | |
| --- | --- | --- | --- |
| Characters |  | Number | Percentage |
| Age | <30 | 27 | 22.5 |
|  | 31-60 | 85 | 70.8 |
|  | >60 | 8 | 6.7 |
| Sex | Male | 88 | 73.3 |
|  | Female | 32 | 26.7 |
| Marital status | Single | 13 | 10.8 |
|  | Married | 107 | 89.2 |
| Educational level (n=105) | None | 37 | 30.8 |
|  | Primary | 29 | 24.2 |
|  | Secondary | 27 | 22.5 |
|  | Tertiary | 12 | 10 |
|  | Other (qur’anic, adult education) | 15 | 12.5 |
| Primary occupation | Farming | 116 | 96.7 |
|  | Trading | 2 | 1.7 |
|  | Civil servant | 2 | 1.7 |
| Years of farming experience | <10 | 14 | 11.7 |
|  | 11-20 | 31 | 25.8 |
|  | 21-30 | 44 | 36.7 |
|  | >30 | 31 | 25.8 |
| Farm size for okra production | ≤2ha | 44 | 36.7 |
|  | 2-3ha | 52 | 43.3 |
|  | >4ha | 24 | 20 |
| Land ownership | Rent/lease | 50 | 41.7 |
|  | Inheritance | 63 | 52.5 |
|  | Purchased | 7 | 5.8 |
| Cultivation cycle/year | Once | 39 | 32.5 |
|  | Twice | 69 | 57.5 |
|  | Thrice | 12 | 10 |
| Cropping system practiced | Mono-cropping | 27 | 22.5 |
|  | Mixed cropping | 93 | 77.5 |
| Type of okra variety grown | Local /landrace | 111 | 92.5 |
|  | Improved (hybrids, OPV) | 9 | 7.5 |
| Sources of seeds | Open market | 35 | 29.2 |
|  | Farmer friend | 20 | 16.7 |
|  | Own saved seed (local) | 63 | 52.5 |
|  | Own saved seed (improved) | 2 | 1.6 |

s

| Supplementary 2: Perception about participatory breeding | | |
| --- | --- | --- |
| N=120 | Yes* | No |
| Inclusion at formulation stage | 111(92.5) | 9(7.5) |
| On-farm trials, evaluation and selection | 116(96.7) | 4(3.3) |
| No involvement | 9(7.5) | 111(92.5) |

*Percentages in parentheses
